# First Description of Marinoquinoline Derivatives’ Activity against *Toxoplasma gondii*

**DOI:** 10.3390/pharmaceutics16020262

**Published:** 2024-02-10

**Authors:** Luiza Tamie Hirata Diethelm, Amanda Bruno da Silva Bellini Ramos, Giovanna Braga de Lorena, Bruna Inácio Trajano, Rafael Dias do Espírito Santo, Renata Priscila Barros de Menezes, Marcus Tullius Scotti, Fabio Antonio Colombo, Marcos José Marques, Carlos Roque Duarte Correia, Juliana Quero Reimão

**Affiliations:** 1 Laboratory of Preclinical Assays and Research of Alternative Sources of Innovative Therapy for Toxoplasmosis and Other Sicknesses (PARASITTOS), Departamento de Morfologia e Patologia Básica, Faculdade de Medicina de Jundiaí, Jundiaí 13202-550, Brazil; 2 Departamento de Análises Clínicas e Toxicológicas, Faculdade de Ciências Farmacêuticas, Universidade Federal de Alfenas, Alfenas 37130-001, Brazil; 1amandabellini@gmail.com (A.B.d.S.B.R.); fabio.colombo@unifal-mg.edu.br (F.A.C.); marques.prppg@gmail.com (M.J.M.); 3 Institute of Chemistry, State University of Campinas, Campinas 13083-970, Brazildiasdoes@ualberta.ca (R.D.d.E.S.); roque@iqm.unicamp.br (C.R.D.C.); 4 Programa de Pós-Graduacão em Produtos Naturais e Sintéticos Bioativos (PgPNSB), Instituto de Pesquisa em Fármacos e Medicamentos (IPeFarM), Universidade Federal da Paraíba, João Pessoa 58051-900, Brazilmtscotti@ccae.ufpb.br (M.T.S.)

**Keywords:** toxoplasmosis, marinoquinolines, anti-*T. gondii* activity, therapeutics, in vitro evaluation, in vivo efficacy, neurotoxoplasmosis

## Abstract

Toxoplasmosis is a globally prevalent zoonotic disease with significant clinical implications, including neurotoxoplasmosis, a leading cause of cerebral lesions in AIDS patients. The current pharmacological treatments for toxoplasmosis face clinical limitations, necessitating the urgent development of new therapeutics. Natural sources have yielded diverse bioactive compounds, serving as the foundation for clinically used derivatives. The exploration of marine bacteria-derived natural products has led to marinoquinolines, which feature a pyrroloquinoline core and demonstrate in vitro and in vivo anti-*Plasmodium* activity. This study investigates the in vitro anti-*Toxoplasma gondii* potential of six marinoquinoline derivatives. Additionally, it conducts absorption, distribution, metabolism, excretion, and toxicity (ADMET) predictions, and evaluates the in vivo efficacy of one selected compound. The compounds displayed half-maximal effective concentration (EC_50_) values between 1.31 and 3.78 µM and half-maximal cytotoxic concentration (CC_50_) values ranging from 4.16 to 30.51 µM, resulting in selectivity indices (SI) from 3.18 to 20.85. MQ-1 exhibiting the highest in vitro SI, significantly reduced tachyzoite numbers in the peritoneum of RH-infected Swiss mice when it was orally administered at 12.5 mg/kg/day for eight consecutive days. Also, MQ-1 significantly reduced the cerebral parasite burden in chronically ME49 infected C57BL/6 mice when it was orally administered at 25 mg/kg/day for 10 consecutive days. These findings underscore the promising anti-*T. gondii* activity of marinoquinolines and their potential as novel therapeutic agents against this disease.

## 1. Introduction

*Toxoplasma gondii* is among the most successful protozoan parasites, causing significant cases of zoonosis globally. While warm-blooded animals are susceptible to infection, only felids serve as definitive hosts for this pathogen. Environmentally resilient oocysts shed by infected felines become infectious after sporulation. Upon ingestion by susceptible hosts, the parasite transforms into tachyzoites, multiplies, and eventually converts into bradyzoites within tissue cysts that are, primarily found in muscle and nerve tissues. Through this mechanism, infection can be transmitted when infected meat is consumed [1].

The prevalence of *T. gondii* infection in humans varies worldwide, with higher rates observed in Central and South America as well as continental Europe, reaching up to 80% in certain locations [2]. In healthy individuals, toxoplasmosis is typically asymptomatic due to immune system action. However, it can cause severe and fatal symptoms in immunocompromised individuals, such as those with Acquired Immunodeficiency Syndrome (AIDS) [3]. An acute infection during pregnancy can be fatal and lead to miscarriage in humans. The risk of mother-to-fetus transmission is from 30% to 45%, with the majority of pregnant humans experiencing subclinical infections (60%). However, 30% of these cases may develop hydrocephaly, intracerebral calcification, chorioretinitis, or mental retardation. Toxoplasmosis can result in fetal death in 9% of congenital infections. The degree of fetal damage depends on the gestational age, with the highest risk occurring early in pregnancy. Although the highest transmission occurs in the third trimester, the fetal lesions are generally less severe during this period [4].

Currently, only a small variety of drugs are effective against this parasitic infection. The combination of sulfadiazine with pyrimethamine is the first choice in the treatment of toxoplasmosis. However, even though it is the best therapeutic option among those available, these drugs are only effective during the active multiplication of the parasite and do not act against the latent phase of the disease, as they do not affect the cysts containing bradyzoites [5]. Therefore, there remains a critical lack of effective drugs for the treatment of the chronic phase of toxoplasmosis, highlighting the pressing need for research into new therapeutic options. Another limitation of the current therapeutic options lies in the wide range of side effects, which can include skin rashes, fever, diarrhea, and allergic reactions, as well as hematological toxicity in some cases [5,6,7].

This study explores the potential of marinoquinoline (MQ) derivatives as a promising class of anti-*Toxoplasma* agents. This class of molecules was first isolated from the bacterium *Rapidithrix thailandica* [8]. All natural MQs feature the 3H-pyrrolo [2,3-c]quinoline core, known as pyrroloquinoline. Some compounds in this class have exhibited in vitro and in vivo activity against *Plasmodium falciparum* [9]. By targeting specific enzymes that are crucial to the parasite’s survival, these compounds offer a strategic avenue for the development of new and more effective treatments against toxoplasmosis. Based on prior knowledge of the anti-*Plasmodium* activity of MQ derivatives, the current study adopts a drug repositioning strategy. This approach offers the advantage of substantially reducing the development time and costs of the treatment compared to traditional methods and has been extensively employed in the drug discovery process for various diseases [10].

Considering the above, one of the objectives of this study was to assess the anti-*T. gondii* effects of MQs. Investigating the anti-*T. gondii* activity of these compounds is relevant and justifiable, and will potentially contribute to the discovery of compounds with action against multiple pathogens.

## 2. Materials and Methods

### 2.1. Synthesis and Characterization of MQs

MQs were prepared according to a previously reported procedure [9].

### 2.2. ADMET Predictions

Prediction of absorption, distribution, metabolism, and excretion (ADME) parameters were calculated using the Swiss ADME open-access web tool (http://www.swissadme.ch (accessed on 18 December 2023)) [11]. The toxicity prediction was performed in the ExplorerDataWarrior v. 4.7.2 software [12] OpenMolecules (https://openmolecules.org/datawarrior/download.html (accessed on 14 January 2024)) and the AdmetSAR server (http://lmmd.ecust.edu.cn/admetsar2/ (accessed on 14 January 2024)) [13,14].

### 2.3. In Vitro Study

#### 2.3.1. Cell Culture and Parasite Propagation

*Toxoplasma gondii* tachyzoites of the RH strain encoding a transgenic β-galactosidase (type I, clone 2F1) [15] were continuously passaged on confluent monolayers of human foreskin fibroblasts (HFFs) cultured in Dulbecco’s Modified Eagle’s Medium (DMEM—Thermo Fisher Scientific, Waltham, MA, USA) supplemented with 2% fetal bovine serum (FBS—Thermo Fisher Scientific) (D2 medium), L-glutamine (2 mM), and gentamicin (10 μg/mL) [16]. Newly emerged tachyzoites were counted, diluted in fresh culture medium, and added to 96-well plates as described below. HFF cell lines and parasites were cultivated in an incubator at 37 °C that was supplemented with 5% CO_2_. *Toxoplasma gondii* cysts of the ME49 strain were obtained from the brain tissue of animals chronically infected with the parasite, following a previously described method [16].

#### 2.3.2. Assay for Evaluating Anti-*T. gondii* Activity

A total of 5 × 10^3^ HFF cells/well (in 100 μL volume) were placed in 96-well plates and incubated over-night to make them adhere. Subsequently, the culture supernatant was aspirated followed by the addition of 5 × 10^3^ tachyzoites of the RH-2F1 strain (in 100 μL volume) and incubation for 3 h at 37 °C, 5% CO_2_. Next, the compounds underwent serial dilution in D2 medium before application to the infected plates, and subsequent incubation occurred over 72 h at 37 °C under 5% CO_2_. β-galactosidase activity assessment followed established protocols [17,18]. Each concentration underwent duplicate evaluation, with pyrimethamine (Sigma-Aldrich, St. Louis, MO, USA) employed as a positive control in all assays.

#### 2.3.3. Assay for Evaluating Mammalian Cytotoxicity

HFF cells were seeded (5 × 10^4^ cells/well) in 96-well microplates and incubated for the following day to make them adhere. Then, the cells were incubated in the presence of increasing compound concentrations for 72 h at 37 °C in a humidified incubator with 5% CO_2_. Cell viability was determined by the 3-[4,5-dimethylthiazol-2-yl]-2,5-diphenyltetrazolium bromide (MTT—Sigma-Aldrich) assay as previously described [18]. Each concentration underwent duplicate evaluation, with pyrimethamine (Sigma-Aldrich) employed as a positive control in all assays. The Selectivity Index (SI) was calculated as the ratio of CC_50_ against HFFs to EC_50_ against *T. gondii*. An SI > 10 was considered promising.

### 2.4. In Vivo Study

Male Swiss mice (*Mus musculus*) (8 weeks old) for the acute toxoplasmosis experiments and female C57BL/6 mice (4 weeks old) for the chronic toxoplasmosis experiments were sourced from the animal breeding facility at the Universidade Federal de Alfenas, Brazil. Strict adherence to the principles of good animal practice, as outlined in the Brazilian Guidelines for Care and Utilization of Animals by the Conselho Nacional de Controle e Experimentação Animal (CONCEA), was observed during the handling of all animals. The study protocol received approval from the Ethics Committee for Animal Experimentation at the Universidade Federal de Alfenas under Protocols 033/2021 and 0006/2023. All necessary precautions were taken to minimize any potential suffering experienced by the animals.

#### 2.4.1. Acute Model

To evaluate the effectiveness of MQ-1 in treating acute *T. gondii* infection in vivo, a murine model was employed, utilizing Swiss mice in accordance with established protocols [19]. These mice underwent intraperitoneal injection with 2 × 10^3^ RH *T. gondii* cultured tachyzoites each and were randomly divided into distinct experimental groups, each consisting of four mice. Treatment involved the administration of compounds dissolved in a 4% DMSO solution in water, and was conducted one day post-infection. The mice received daily oral doses of either MQ-1 or PYR at 12.5 mg/kg for a duration of 8 consecutive days. As for the control group, they were administered 100 µL of the vehicle utilized for MQ-1 dilution, consisting of a 4% DMSO solution in water. Continuous monitoring of the mice included daily assessments for signs of toxoplasmosis. These signs encompassed impaired mobility, feeding difficulties, weight loss, self-mutilation, and severe ascites. Humane endpoints were implemented when mice displayed the afore mentioned signs, maintained the maximum tolerable score for two consecutive days, and were deemed beyond the possibility of recovery. The implementation of humane endpoints aimed to mitigate any potential pain or suffering in the animals, leading to their euthanasia when necessary [20]. At the end of the experimental period, euthanasia was performed on all remaining mice, and the peritoneal parasite load was estimated using a Neubauer chamber.

#### 2.4.2. Chronic Model

The mice were infected with 10 cysts of the ME49 strain of *T. gondii* via intraperitoneal inoculation, following the protocol by [16]. After a 40-day infection period, random assignment was used to divide the mice into distinct experimental groups, each consisting of four mice. These experimental groups were subjected to different treatments: MQ-1, administered either at a dosage of 25 mg/kg/day or 12.5 mg/kg/day, prepared with a 4% DMSO solution in water. Additionally, another group received pyrimethamine at a dosage of 12.5 mg/kg/day, prepared using distilled water. The administration was carried out via oral gavage, with a final volume of 100 µL per dose. For the control group, 100 µL of the vehicle used to dilute MQ-1 (4% DMSO in water) was administered. The selection of the 12.5 mg/kg/day pyrimethamine dose was based on a previous study [21]. To facilitate a direct comparison of efficacy between the two compounds, the decision was made to use the same dose of 12.5 mg/kg/day for MQ-1 and also to employ a double dose of 25 mg/kg/day. On the 51st day post-infection, euthanasia was performed for all animals, followed by brain tissue collection for RNA extraction and quantification, as detailed in [18]. DNA extraction and purification were carried out using the PureLink Genomic DNA Minikit (Invitrogen. Carlsbad, CA, USA) following the manufacturer’s instructions. For RNA extraction and purification from the brain tissue, the Purelink RNA Minikit was employed, and the manufacturer’s instructions were closely adhered to during the process. After extraction, RNA samples were immediately processed for cDNA synthesis and underwent quantitative real-time (qPCR) analysis. The qPCR was executed, and the cycle threshold values (C_T_) were correlated with the parasite count, utilizing a standard curve method as previously described in [18].

### 2.5. Statistical Analysis

Data on parasite burden in acute and chronic infection in treated and untreated groups were analyzed for statistical significance using One-Way ANOVA, followed by the Tukey post-test. Statistical analyses were performed using GraphPad Prism 9 software. A result was considered significant at *p* < 0.05.

## 3. Results

### 3.1. Chemical Characterization of MQs Derivatives Evaluated against T. gondii

**Figure 1:**
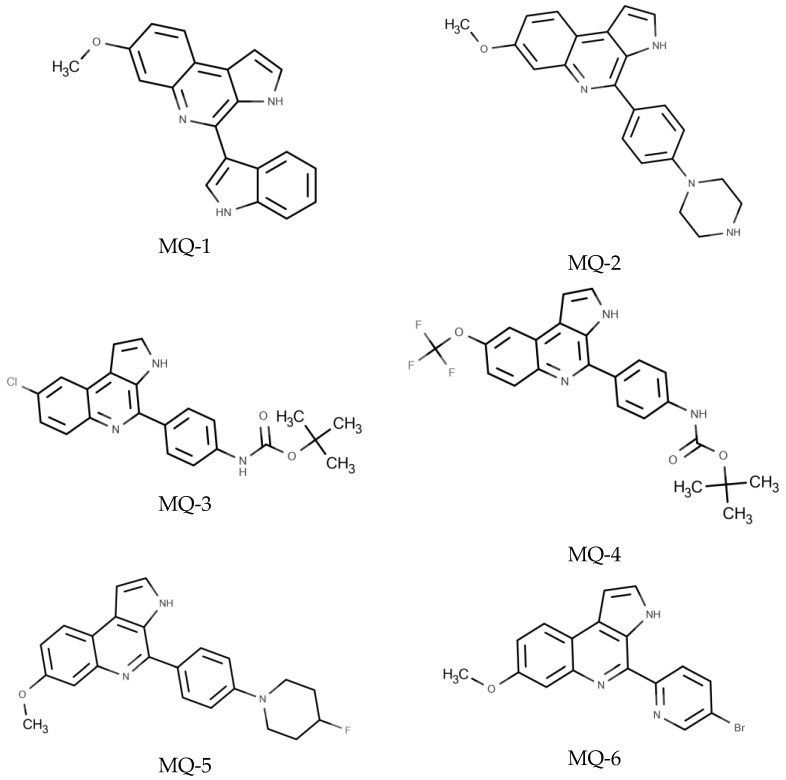
Chemical structures of marinoquinoline derivatives evaluated against *T. gondii*.

The MQ-1 chemical characterization was reported by [9]. The ^1^H and ^13^C NMR characterizations of MQ-2 to MQ-6 are shown in the Supplementary Materials. The chemical structures of the compounds under study are shown in Figure 1.

### 3.2. In Silico Results

#### ADMET Predictions

Initially, predictions of the absorption, distribution, metabolism, excretion, and toxicity (ADMET) were performed using the Swiss ADME, DataWarrior and admetSAR platforms. The objective of the analysis was to identify compounds with favorable pharmacokinetic, pharmacochemical, and pharmacological profiles, thus indicating their potential for further development. Information regarding gastrointestinal absorption, blood–brain barrier permeability, mutagenicity, tumorigenicity, and reproductive effects is presented in Table 1.

The potential interactions between the six MQs under examination and the reference drug pyrimethamine were investigated, considering the five major cytochrome P450 isoforms (CYP1A2, CYP2C19, CYP2C9, CYP2D6, CYP3A4) (Table 2). Interestingly, all MQ derivatives were predicted to inhibit most of these CYP isoforms. Only MQ-1, MQ-2, MQ-5, and the reference drug showed no predicted inhibitory effect on CYP2C9. Furthermore, MQ-2 was not predicted to inhibit CYP2C19.

**Figure 2:**
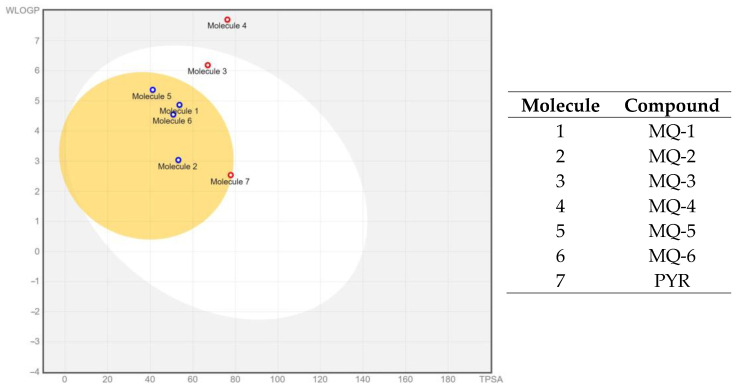
Boiled Egg diagram of the six marinoquinoline derivatives under study. Points located in the yellow area indicate molecules predicted to passively permeate the blood-brain barrier. Blue points indicate molecules predicted to be effluxed from the central nervous system by P-glycoprotein. Red points indicate molecules predicted not to be effluxed from the central nervous system by P-glycoprotein. Obtained from Swiss ADME web tool. (www.swissadme.ch (accessed on 18 December 2023)).

The gastrointestinal absorption and brain penetration of the six MQ derivatives under investigation, as well as the standard drug (pyrimethamine), were illustrated using a Boiled Egg diagram, as depicted in Figure 2, employing the Swiss ADME web tool. The white region in the diagram represents the area with the highest likelihood of absorption by the human gastrointestinal system, while the yellow portion (the yolk) represents the region with the highest probability of brain penetration. The diagram revealed that most of the compounds under examination, including the standard drug, fall within the yellow region (yolk), except for molecules 3 and 4 (MQ-3 and MQ-4). This suggests that four MQ derivatives (MQ-1, MQ-2, MQ-5, and MQ-6) exhibit a high probability of being absorbed by the human gastrointestinal tract and permeating the brain to reach *T. gondii* cysts in the brain.

Understanding whether compounds act as substrates or non-substrates of the permeability glycoprotein is crucial for assessing the active efflux across biological membranes, such as the blood–brain barrier. Figure 2 reveals that only two MQ derivatives (MQ-3 and MQ-4) were predicted as non-substrates of P-glycoprotein and thus not effluxed from the central nervous system (CNS).

### 3.3. In Vitro Results

**Figure 3:**
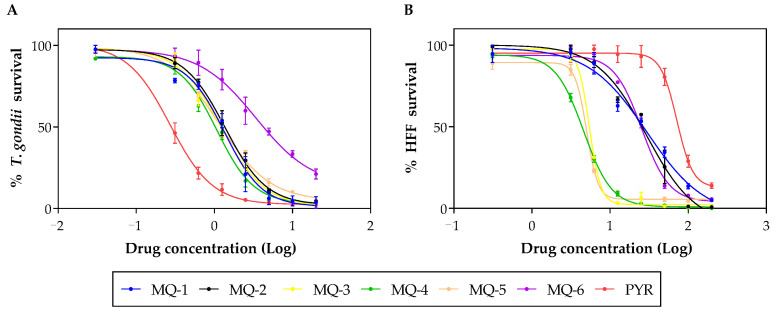
Representative dose-response curves for the six marinoquinoline derivatives and pyrimethamine against *T. gondii* (**A**) and HFF (**B**).

The biological activity of the six MQ derivatives was assessed against a human cell line (HFF) and *T. gondii* tachyzoites from the virulent RH strain. The CC_50_ values, indicating toxicity against human cells, and EC_50_ values, indicating anti-parasitic activity, were obtained through the analysis of the dose-response curves (Figure 3).

By calculating the ratio between the CC_50_ and EC_50_, SI values were determined, as shown in Table 3. A higher SI value suggests a more promising compound, indicating a more selective anti-parasitic action. As a result, MQ-1 emerged as the most promising MQ derivative among the six that were evaluated. Pyrimethamine was used as a reference drug and its obtained EC_50_ value was consistent with previously reported values [22]. Only MQ-1, MQ-2 and pyrimethamine presented SI > 10.

### 3.4. In Vivo Results

Considering the in silico and in vitro findings, particularly the predictions of high gastrointestinal absorption, the absence of toxicity (tumorigenicity, mutagenicity, and reproductive effects), and a higher SI, the compound MQ-1 was selected from among the six MQ derivatives under investigation.

**Figure 4:**
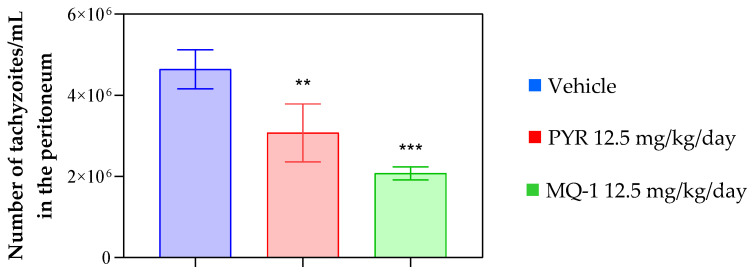
Effect of MQ-1 on an animal model of acute toxoplasmosis. Swiss mice were intraperitoneally (ip) infected with 2 × 10^3^ RH strain tachyzoites. Starting one day post-infection, MQ-1 was administered orally at 12.5 mg/kg/day, the vehicle (4% DMSO) was administered at the same rate, or the positive control drug pyrimethamine (PYR) was administered at 12.5 mg/kg/day for 8 consecutive days (*n* = 4 for each group). Effect of MQ-1 on parasite burden in the peritoneum of infected mice, estimated by microscopic counting. Asterisks indicates significant differences in comparison with the vehicle group (** *p* < 0.01; *** *p* < 0.001).

Initially, the Swiss mice infected with the virulent RH strain were treated with the compound MQ-1 or the standard drug pyrimethamine at a dose of 12.5 mg/kg/day for 8 days. At the end of treatment, the parasite burden was estimated by microscopic counting of the tachyzoites harvested from the peritoneal cavity. As observed in Figure 4, there was a significant reduction (*p* < 0.001) in the number of tachyzoites/mL collected from the peritoneum of the animals that received treatment with MQ-1 compared to the untreated control. No signs of toxicity were observed throughout the treatment.

**Figure 5:**
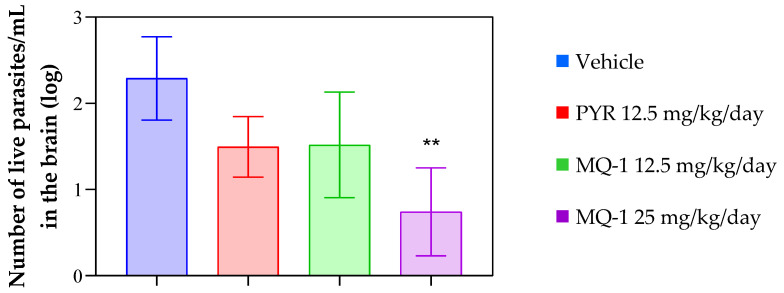
Effect of MQ-1 on an animal model of chronic toxoplasmosis. C57BL/6 mice were intraperitoneally (ip) infected with 10 ME49 strain brain cysts. Starting 41 days post-infection, MQ-1 was administered orally at 12.5 or 25 mg/kg/day, the vehicle (4% DMSO) was administered, or the positive control drug pyrimethamine (PYR) was administered at 12.5 mg/kg/day for 10 consecutive days (*n* = 4 for each group). These mice were euthanized on day 51 post-infection, and their brains were collected for RNA quantification. Number of live parasites/mL estimated by qPCR. Asterisks indicates significant differences in comparison with the vehicle group (** *p* < 0.01).

Due to the necessity of researching drugs that are effective against the chronic form of toxoplasmosis and considering the predicted blood-brain barrier permeability of the compound MQ-1, it was decided to evaluate the efficacy of this compound in an experimental model of chronic toxoplasmosis. For this purpose, C57BL/6 mice infected with the ME49 *T. gondii* cystogenic strain were used. The treatment began 41 days after infection, when the toxoplasmosis had already become chronic and generated brain cysts. The animals received daily oral doses of MQ-1 at 12.5 and 25 mg/kg/day for 10 days, and the treatment’s effect was compared with the standard drug and a group that did not receive pharmacological treatment. As observed in Figure 5, treatment with the higher dose of the compound MQ-1 led to a significant (*p* < 0.01) reduction in the cerebral parasitic load of the chronically infected mice, indicating that this compound can reach therapeutic doses in the brain to exert its anti-*T. gondii* action on the characteristic cysts of chronic infection. The results showed no statistically significant differences between the tested compound and the standard drug in both acute and chronic evaluations.

## 4. Discussion

This study is the first description of the anti-*T. gondii* activity exhibited by compounds belonging to the MQ class. Marinoquinolines are known for their biological activity against *Plasmodium* [9], the causative agent of malaria, which, like *T. gondii*, falls within the grouping of apicomplexan protozoa [23]. The presented findings corroborate the anti-parasitic activity of MQ derivatives and suggest a possible biological activity against other apicomplexan parasites. Numerous examples exist of compounds displaying biological activity against both *Toxoplasma* and *Plasmodium* parasites, as demonstrated by clinically used drugs such as artemisinin and atovaquone [6].

In the initial stage of this investigation, in silico tools played a crucial role in predicting the pharmacokinetic and pharmacodynamic properties of six MQ derivatives. These predictions aimed to identify compounds with favorable profiles for further examination. The ADME predictions of bioactive compounds in the early stages of drug development have been instrumental in guiding researchers toward selecting candidates with optimal pharmacokinetic profiles [24]. The utilization of these computational tools allowed for a comprehensive assessment of the compounds’ potentials, guiding the selection of candidates for subsequent in vitro and in vivo evaluations [25].

Notably, MQ-1, MQ-2, MQ-5, and MQ-6 exhibited high predicted gastrointestinal absorption, a pivotal determinant for assessing oral bioavailability, as previously highlighted in studies on drug development [26]. Moreover, these compounds demonstrated significant potential for penetrating the blood-brain barrier, a crucial factor when dealing with CNS-related infections, as previously demonstrated in investigations of CNS-permeable drugs [27]. Intriguingly, MQ-1, MQ-2, MQ-5, and MQ-6 exhibited no predicted inhibitory effects on CYP2C9, a key cytochrome P450 isoform, suggesting a reduced likelihood of drug-drug interactions, as suggested by [28].

Additionally, the Boiled Egg diagram offered valuable insights into the compounds’ passive permeation potential across the blood–brain barrier, as demonstrated in previous studies which evaluated the CNS penetration of pharmaceutical agents [29]. Most of the MQ derivatives displayed a high probability of CNS penetration, positioning them as promising candidates for addressing CNS-involved infections.

The therapeutic potential of 3H-pyrrolo [2,3-c]quinolines, the core structure of MQs natural products, as anti-Tuberculosis agent was previously explored [30]. Two derivatives, compounds 50 and 54, derived from this scaffold, exhibited significant in vitro activity against virulent strains of *Mycobacterium tuberculosis*. Enzymatic assays identified them as inhibitors of glutamate-5-kinase (G5K), a pivotal enzyme encoded by the proB gene. G5K plays a crucial role in the proline biosynthesis pathway, catalyzing the phosphoryl-transference of the γ-phosphate group from ATP to L-glutamate, yielding L-glutamyl-5-phosphate and ADP. Additionally, G5K serves as a key regulator in L-proline synthesis. Notably, these findings led us to hypothesize that G5K could potentially be a target for the action of MQs against *T. gondii*; however, further studies are required to validate this hypothesis.

The significance of G5K in *Leishmania* parasites was investigated by [31], establishing its catalytic function as a G5K and its allosteric regulation by proline. Their findings emphasize the essential role of G5K in proline biosynthesis, which is crucial for the parasite’s survival. Similarly, the crystal structures of G5K from *Escherichia coli* were explored by [32], revealing a tetrameric architecture with unique features. This includes the absence of a C-terminal PUA domain and a non-canonical association between AAK and PUA domains in the dimeric structure. The insights from both studies contribute to our understanding of G5K, providing a broader context for its potential as a target. While our study focuses on the anti-*Toxoplasma* activity of MQs, the parallels drawn between these works strengthen the rationale for investigating G5K as a putative target for therapeutic intervention against protozoan parasites, opening avenues for future exploration and validation.

Furthermore, it is essential to highlight that the compounds were also assessed for selectivity concerning their cytotoxicity against human fibroblasts. This cell line serves as a common choice in in vitro studies assessing anti-*T. gondii* activity, serving as a host for the parasite [16]. The compounds with lower cytotoxicity were ranked as follows: MQ-1, MQ-2, MQ-6, MQ-3, MQ-5, and MQ-4. As a result, MQ-1 and MQ-2 exhibited the highest SI, indicating significant potential for specific action against *T. gondii* tachyzoites. The SI has been considered a crucial parameter in evaluating the therapeutic potential of anti-parasitic agents [33].

Additionally, it is noteworthy that the compound MQ-4 exhibited unfavorable predictions concerning gastrointestinal absorption. This characteristic suggests that MQ-4 may not be an ideal candidate for future in vivo evaluation, particularly when considering the oral route of administration. Compounds with a low predicted gastrointestinal absorption may fail to exhibit an anti-parasitic effect when orally administered in infected animals, as indicated in prior studies [24].

Considering these findings, MQ-1 has emerged as a promising choice for in vivo assessments. It demonstrated both high in vitro anti-*T. gondii* activity and low cytotoxicity against human fibroblasts, along with favorable predictions of its absorption and CNS permeability. Previous studies applying the same rationale have been successful in discovering compounds with efficacy against chronic toxoplasmosis [18,34].

Therefore, in vivo experiments were conducted employing Swiss mice infected with the RH strain as an experimental model of acute toxoplasmosis. The administration of Type I strains through the intraperitoneal (IP) injection of tachyzoites, as employed in this study, allows for a consistent and controlled induction of acute infection, facilitating the evaluation of a compound’s therapeutic efficacy against *T. gondii* [16].

MQ-1 showed efficacy against acute *T. gondii* infection, as demonstrated by the reduction in the number of tachyzoites in the peritoneum of RH-infected mice. To further evaluate its potential clinical relevance, additional studies assessing the compound’s safety profile, long-term effects, and potential resistance mechanisms should be considered in future investigations.

Also, the experimental treatment employed the ME49 strain, a type II *T. gondii* strain characterized by inducing chronic infection in C57BL/6 mice, resulting in the formation of brain cysts replete with bradyzoites [16]. Bradyzoites enclosed within brain cysts are notoriously challenging to eliminate from the host [35]. Consequently, the identification of a new class of compounds capable of reducing brain cysts in susceptible animals represents a highly relevant discovery within the realm of drug research and development for toxoplasmosis [36].

In a prior study, MQ derivatives were found to display inhibitory activity against ring forms of *P. falciparum* within a range from 0.6 to 0.039 µM, and the most potent compound exhibited significant selectivity (SI > 6410) [9]. This elevated SI stands in contrast to the SI observed in the current study with *T. gondii*. It is noteworthy that the earlier investigation involved a quantitative structure–activity relationship (QSAR) study, facilitating the discovery of analogs with enhanced potency. Future studies could strategically design derivatives with improved activity against *T. gondii*, aiming to achieve higher SI values.

Moreover, the identification of compounds with potential for CNS penetration and selective anti-*T. gondii* activity opens avenues for additional research. Future studies that assess other compounds within the MQ class would be desirable, including those aimed at the rational design of novel analogs with enhanced activity against the parasite. Evaluating additional compounds with diverse physicochemical properties may provide fresh insights into the structure-activity relationship of this compound class against *T. gondii*. Moreover, MQ-1 can serve as a prototype for the development and refinement of new molecules, thus contributing to the discovery of more effective and less toxic anti-*T. gondii* compounds. Future research efforts can delve deeper into elucidating the exact mode of action of MQs against *T. gondii*, potentially unveiling novel targets for therapeutic intervention.

This study marks a significant step forward in understanding the potential of MQ derivatives as anti-*T. gondii* agents. These compounds, previously recognized for their anti-*Plasmodium* [9] and anti-*Mycobacterium* [30] activities, have demonstrated a promising avenue for combating *T. gondii* experimental infection. Importantly, the presented findings suggest a shared target between these pathogens, paving the way for the further exploration of MQs as drug candidates.

## 5. Conclusions

In summary, this study reveals the potential of MQ derivatives as anti-*T. gondii* agents, indicating a possible shared mechanism of action with other pathogens. The selection of compound MQ-1 as a promising candidate for in vivo studies and the demonstration of its efficacy against acute and chronic *T. gondii* infection represent significant advancements in the research of new treatments for toxoplasmosis. However, it is important to acknowledge that there is room for future investigations and enhancements, including the exploration of other MQ derivatives and a more in-depth investigation of the mechanism of action of these compounds against *T. gondii*. This study marks a significant step in the path towards the development of novel therapeutic agents against this infection.

## Figures and Tables

**Table 1:** Main results of ADMET predictions for the six marinoquinoline derivatives and pyrimethamine.

Compound	GI ^1^	BBB ^1^	Mut ^2^	Tum ^2^	RE ^2^	AOT ^3^	RT ^4^	SC ^4^	SI ^4^	SS ^4^	MT ^4^	NT ^4^
MQ-1	+	+	−	−	−	−	−	−	−	−	−	−
MQ-2	+	+	−	−	−	−	+	−	−	−	+	−
MQ-3	+	−	−	−	−	−	−	−	−	−	−	+
MQ-4	−	−	−	−	−	−	+	−	−	−	−	+
MQ-5	+	+	−	−	−	−	+	−	−	−	+	−
MQ-6	+	+	−	−	−	−	+	−	−	−	−	−
PYR ^5^	+	+	+	+	+	+	+	−	−	−	+	+

^1^ Gastrointestinal absorption (GI) and blood-brain barrier permeation (BBB) were generated by Swiss ADME web tool (www.swissadme.ch (accessed on 18 December 2023)). ^2^ Mutagenicity (Mut), tumorigenicity (Tum), and reproductive effect (RE) were predicted by DataWarrior software v. 4.7.2. ^3^ Acute oral toxicity (AOT), classified into four categories based on the Half Lethal Dose (LD_50_): positive (+) (LD_50_ ≤ 50, >50 mg/kg) and negative (−) (LD_50_ > 500 and >5000 mg/kg), as predicted by AdmetSAR server. ^4^ Respiratory toxicity (RT), skin corrosion (SC), skin irritation (SI), mitochondrial toxicity (MT) and nephrotoxicity (NT) were predicted by AdmetSAR server. ^5^ Pyrimethamine (PYR) was used as reference drug.

**Table 2:** Predicted interaction with cytochromes P450 (CYP) for six marinoquinoline derivatives and pyrimethamine.

Compound	CYP ^1^				
	CYP1A2	CYP2C19	CYP2C9	CYP2D6	CYP3A4
MQ-1	Yes	Yes	No	Yes	Yes
MQ-2	Yes	No	No	Yes	Yes
MQ-3	Yes	Yes	Yes	Yes	Yes
MQ-4	Yes	Yes	Yes	Yes	Yes
MQ-5	Yes	Yes	No	Yes	Yes
MQ-6	Yes	Yes	Yes	Yes	Yes
PYR ^2^	Yes	Yes	No	No	Yes

^1^ Predicted interaction with cytochromes P450 (CYP) and five major isoforms were generated by Swiss ADME web tool (www.swissadme.ch (accessed on 18 December 2023)). ^2^ Pyrimethamine (PYR) was used as reference drug.

**Table 3:** Selectivity of marinoquinoline derivatives against *T. gondii*: EC_50_, CC_50_, and Selectivity Index (SI).

Compound	CC_50_ (µM) ^1^	EC_50_ (µM) ^2^	SI ^3^
MQ-1	30.51 ± 1.80	1.46 ± 0.45	20.85
MQ-2	29.11 ± 0.84	1.67 ± 0.40	17.39
MQ-3	5.36 ± 0.91	1.60 ± 0.54	3.35
MQ-4	4.16 ± 1.05	1.31 ± 0.31	3.18
MQ-5	5.05 ± 1.63	1.43 ± 0.37	3.52
MQ-6	28.55 ± 7.33	3.78 ± 0.67	7.55
PYR ^4^	68.11 ± 4.26	0.29 ± 0.19	237.65

^1^ Half Cytotoxic Concentration (CC_50_) against HFF cells ± Standard Deviation of three independent experiments. ^2^ Half Effective Concentration (EC_50_) against *T. gondii* tachyzoites ± Standard Deviation of three independent experiments. ^3^ Selectivity index (SI), calculated based on CC_50_/EC_50_. ^4^ Pyrimethamine (PYR) was used as reference drug.

## Data Availability

The data presented in this study are available in this article and Supplementary Material.

## Supplementary Materials

The following supporting information can be downloaded at: https://www.mdpi.com/article/10.3390/pharmaceutics16020262/s1, Table S1: Spectroscopic and spectrometric characterization of MQ-2 to MQ-6; Table S2: ADMET properties predictions for six marinoquinoline derivatives and pyrimethamine according to SwissModel; Table S3: ADMET properties predictions for six marinoquinoline derivatives and pyrimethamine according to AdmetSAR.
