# Supplementary material for: First Description of Marinoquinoline Derivatives’ Activity against Toxoplasma gondii
Source: Pharmaceutics. 2024 Feb 10;16(2):262. doi: 10.3390/pharmaceutics16020262 (PMC10891983; doi:10.3390/pharmaceutics16020262)
Supplement: Supplementary file 1 [file pharmaceutics-16-00262-s001.zip › pharmaceutics-2800147-supplementary.pdf]

pharmaceutics

MDPI

*Supplementary Materials*

# First description of marinoquinoline derivatives' activity against *Toxoplasma gondii*

**Luiza Tamie Hirata Diethelm [1], Amanda Bruno da Silva Bellini Ramos [2], Giovanna Braga de Lorena [1], Bruna Inácio Trajano [3], Rafael Dias do Espírito Santo [3], Renata Priscila Barros de Menezes [4], Marcus Tullius Scotti [4], Fabio Antonio Colombo [2], Marcos José Marques [2], Carlos Roque Duarte Correia [3] and Juliana Quero Reimão [1,*]**

1 Laboratory of Preclinical Assays and Research of Alternative Sources of Innovative Therapy for Toxoplasmosis and Other Sicknesses (PARASITTOS), Departamento de Morfologia e Patologia Básica, Faculdade de Medicina de Jundiaí, Jundiaí 13202-550, Brazil

2 Departamento de Análises Clínicas e Toxicológicas, Faculdade de Ciências Farmacêuticas, Universidade Federal de Alfenas, Alfenas 371300-001, Brazil; 1amandabellini@gmail.com (A.B.d.S.B.R.); fabio.colombo@unifal-mg.edu.br (F.A.C.); marques.prppg@gmail.com (M.J.M.)

3 Institute of Chemistry, State University of Campinas, Campinas 13083-970, Brazil; diasdoes@ualberta.ca (R.D.d.E.S.); roque@iqm.unicamp.br (C.R.D.C.)

4 Programa de Pós-Graduacão em Produtos Naturais e Sintéticos Bioativos (PgPNSB), Instituto de Pesquisa em Fármacos e Medicamentos (IPeFarM), Universidade Federal da Paraíba, João Pessoa 58051-900, Brazil; mtscotti@ccae.ufpb.br (M.T.S.)

* Correspondence: julianareimao@g.fmj.br

**Supplementary Material Table S1.** Spectroscopic and spectrometric characterization of MQ-2 to MQ-6.

### MQ-2

7-methoxy-4-(4-(piperazin-1-yl)phenyl)-3H-pyrrolo[2,3-c]quinoline

Yellow solid. M.P.: 206-207 °C.

$^{1}$H NMR (500 MHz, DMSO) δ 11.68 (s, 1H), 8.16 (d, J = 8.9 Hz, 1H), 8.00 – 7.91 (m, 2H), 7.55 (d, J = 3.0 Hz, 1H), 7.46 (d, J = 2.6 Hz, 1H), 7.17 (dd, J = 8.8, 2.6 Hz, 1H), 7.14 – 7.06 (m, 3H), 3.90 (s, 3H), 3.25 – 3.16 (m, 4H), 2.95 – 2.89 (m, 4H).

$^{13}$C NMR (126 MHz, DMSO) δ 157.6, 151.9, 146.1, 143.6, 129.5, 129.3, 128.4, 128.1, 125.9, 124.0, 117.0, 116.4, 114.8, 108.7, 100.6, 55.2, 48.4, 45.2.

HRMS (ESI+) m/z calculated for $[C_{22}H_{23}N_4O^+]$: 359.1866. Found: 359.1867.

### MQ-3

tert-butyl (4-(8-chloro-3H-pyrrolo[2,3-c]quinolin-4-yl)phenyl)carbamate

Amorphous orange solid.

$^{1}$H NMR (400 MHz, DMSO) δ 11.92 (s, 1H), 9.62 (s, 1H), 8.39 (d, J = 2.4 Hz, 1H), 8.04 (d, J = 8.9 Hz, 1H), 7.98 – 7.90 (m, 2H), 7.72 – 7.67 (m, 2H), 7.66 – 7.61 (m, 1H), 7.55 (dd, J = 8.9, 2.5 Hz, 1H), 7.29 (d, J = 2.9 Hz, 1H), 1.51 (s, 9H).

$^{13}$C NMR (101 MHz, DMSO) δ 152.8, 146.4, 140.8, 140.6, 131.4, 131.0, 129.7, 129.2, 128.5, 128.3, 126.8, 126.1, 123.9, 122.2, 118.1, 101.7, 79.4, 28.2.

HRMS (ESI+) m/z calculated for $[C_{22}H_{21}ClN_3O_2^+]$: 394.1317. Found: 394.1322.

### MQ-4

tert-butyl (4-(8-(trifluoromethoxy)-3H-pyrrolo[2,3-c]quinolin-4-yl)phenyl)carbamate

Amorphous white solid.

$^{1}$H NMR (400 MHz, Acetone) δ 11.46 (s, 1H), 8.77 (s, 1H), 8.26 – 8.17 (m, 2H), 8.03 – 7.98 (m, 2H), 7.77 – 7.70 (m, 2H), 7.67 – 7.61 (m, 1H), 7.50 (dd, J = 9.8, 2.4 Hz, 1H), 7.26 (d, J = 3.0 Hz, 1H), 1.50 (s, 9H).

$^{13}$C NMR (63 MHz, DMSO) δ 153.2, 147.2, 145.9 (q, J = 1.9 Hz), 141.3, 141.2, 131.9, 131.8, 129.7, 129.2, 129.0, 127.2, 123.8, 120.8 (q, J = 256.0 Hz), 119.6, 118.5, 115.1, 102.2, 79.8, 28.6.

HRMS (ESI+) m/z calculated for $[C_{23}H_{21}F_3N_3O_3^+]$: 444.1530. Found: 444.1540.

(cont.)

| MQ-5 |
| --- |
| 4-(4-(4-fluoropiperidin-1-yl)phenyl)-7-methoxy-3H-pyrrolo[2,3-c]quinoline<br>Amorphous yellow solid.<br>$^{1}H$ NMR (500 MHz, Acetone) δ 11.02 (s, 1H), 8.17 (d, J = 8.8 Hz, 1H), 8.04 – 7.97 (m, 2H), 7.60 (d, J = 2.9 Hz, 1H), 7.56 (d, J = 2.6 Hz, 1H), 7.18 (dd, J = 8.8, 2.6 Hz, 1H), 7.17 – 7.11 (m, 2H), 7.11 (d, J = 2.9 Hz, 1H), 4.86 (dtt, J = 49.1, 7.1, 3.5 Hz, 1H), 3.94 (s, 3H), 3.59 – 3.49 (m, 2H), 3.30 (ddd, J = 12.9, 7.4, 3.7 Hz, 2H), 2.13 – 2.05 (m, 2H), 1.95 – 1.82 (m, 2H).<br>$^{13}C$ NMR (126 MHz, Acetone) δ 159.1, 152.4, 147.2, 145.0, 130.6, 130.5, 129.6, 128.4, 127.4, 124.7, 118.3, 117.5, 116.3, 109.5, 101.5, 89.4 (d, J = 170.3 Hz), 55.6, 45.8 (d, J = 6.9 Hz), 31.8 (d, J = 19.3 Hz).<br>HRMS (ESI+) m/z calculated for [$C_{23}H_{23}FN_3O^+$]: 376.1820. Found: 376.1806. |
| **MQ-6** |
| 4-(5-bromopyridin-2-yl)-7-methoxy-3H-pyrrolo[2,3-c]quinoline<br>[MQ-6]<br>Yellow solid. M.P. 187-188 °C.<br>$^{1}H$ NMR (500 MHz, DMSO) δ 12.01 (s, 1H), 8.90 (d, J = 2.2 Hz, 1H), 8.70 (d, J = 8.6 Hz, 1H), 8.32 (dd, J = 8.6, 2.4 Hz, 1H), 8.25 (d, J = 8.9 Hz, 1H), 7.72 – 7.67 (m, 1H), 7.56 (d, J = 2.6 Hz, 1H), 7.28 (dd, J = 8.9, 2.6 Hz, 1H), 7.17 – 7.13 (m, 1H), 3.94 (s, 3H).<br>$^{13}C$ NMR (126 MHz, DMSO) δ 157.8, 155.3, 149.3, 142.8, 140.7, 140.0, 130.3, 128.9, 126.0, 124.2, 123.4, 120.7, 118.3, 118.2, 108.7, 100.0, 55.2.<br>HRMS (ESI+) m/z calculated for [$C_{17}H_{13}BrN_3O^+$]: 354.0237. Found: 354.0235. |

**Supplementary Material Table S2.** ADMET properties predictions for six marinoquinoline derivatives and pyrimethamine according to SwissModel.

| | | MQ-1 | MQ-2 | MQ-3 | MQ-4 | MQ-5 | MQ-6 | PYR[1] |
|---|---|---|---|---|---|---|---|---|
| **Physicochemical properties** | Formula Molecular | $C_{20}H_{15}N_3O$ | $C_{22}H_{22}N_4O$ | $C_{22}H_{20}ClN_3O_2$ | $C_{23}H_{20}F_3N_3O_3$ | $C_{23}H_{22}FN_3O$ | $C_{17}H_{12}BrN_3O$ | $C_{13}H_8Cl_2N_2O_4$ |
| | Molecular Weight | 313.35 | 358.44 | 393.87 | 443.42 | 375.44 | 354.2 | 248.71 |
| | #Heavy atoms | 24 | 27 | 28 | 32 | 28 | 22 | 17 |
| | #Aromatic heavy atoms | 22 | 19 | 19 | 19 | 19 | 19 | 12 |
| | Fraction Csp3 | 0.05 | 0.23 | 0.18 | 0.22 | 0.26 | 0.06 | 0.17 |
| | #Rotatable bonds | 2 | 3 | 5 | 7 | 3 | 2 | 2 |
| | #H-bond acceptors | 2 | 3 | 3 | 7 | 3 | 3 | 2 |
| | #H-bond donors | 2 | 2 | 2 | 2 | 1 | 1 | 2 |
| | MR | 97.38 | 116.89 | 114.29 | 115.96 | 115.03 | 91.02 | 71.06 |
| | TPSA[2] | 53.7 | 53.18 | 67.01 | 76.24 | 41.15 | 50.8 | 77.82 |
| **Lipophilicity** | iLOGP | 2.46 | 3.06 | 3.76 | 3.88 | 3.27 | 3.31 | 2.15 |
| | XLOGP3 | 4.08 | 3.42 | 5.17 | 5.72 | 4.97 | 3.61 | 2.69 |
| | WLOGP | 4.87 | 3.04 | 6.19 | 7.7 | 5.37 | 4.55 | 2.54 |
| | MLOGP | 2.32 | 2.08 | 3.44 | 2.94 | 3.23 | 2.12 | 1.64 |
| | Silicos-IT Log P | 5.08 | 4.12 | 5.14 | 5.13 | 5.07 | 4.66 | 2.44 |
| | Consensus Log P | 3.76 | 3.14 | 4.74 | 5.07 | 4.38 | 3.65 | 2.29 |
| **Water solubility** | ESOL Log S | -4.9 | -4.54 | -5.71 | -6.17 | -5.6 | -4.82 | -4.9 |
| | ESOL Solubility (mg/ml) | 3.95e-03 | 1.03e-02 | 7.66e-04 | 3.00e-04 | 9.37e-04 | 5.39e-03 | 0.00395 |
| | ESOL Solubility (mol/l) | 1.26e-05 | 2.89e-05 | 1.94e-06 | 6.76e-07 | 2.49e-06 | 1.52e-05 | 0.0000126 |
| | ESOL Class | MS[3] | MS | MS | PS[4] | MS | MS | MS |
| | Ali Log S | -4.91 | -4.22 | -6.32 | -7.09 | -5.57 | -4.36 | -4.91 |
| | Ali Solubility (mg/ml) | 3.83e-03 | 2.18e-02 | 1.87e-04 | 3.62e-05 | 1.00e-03 | 1.53e-02 | 0.00383 |
| | Ali Solubility (mol/l) | 1.22e-05 | 6.07e-05 | 4.75e-07 | 8.17e-08 | 2.67e-06 | 4.32e-05 | 0.0000122 |
| | Ali Class | MS | MS | PS | PS | MS | MS | MS |
| | Silicos-IT LogSw | -8.1 | -7.68 | -8.6 | -8.56 | -7.95 | -7.67 | -8.1 |
| | Silicos-IT Solubility (mg/ml) | 2.49e-06 | 7.44e-06 | 9.94e-07 | 1.21e-06 | 4.25e-06 | 7.61e-06 | 0.00000249 |
| | Silicos-IT Solubility (mol/l) | 7.94e-09 | 2.07e-08 | 2.52e-09 | 2.74e-09 | 1.13e-08 | 2.15e-08 | 7.94E-09 |
| | Silicos-IT class | PS | PS | PS | PS | PS | PS | MS |

(cont.)

| | | | | | | | | |
|---|---|---|---|---|---|---|---|---|
| **Pharmacokinetics properties** | GI[5] absorption | High | High | High | Low | High | High | High |
| | BBB[6] permeant | Yes | Yes | No | No | Yes | Yes | Yes |
| | Pgp[7] substrate | Yes | Yes | No | No | Yes | Yes | No |
| | CYP1A2 inhibitor | Yes | Yes | Yes | Yes | Yes | Yes | Yes |
| | CYP2C19 inhibitor | Yes | No | Yes | Yes | Yes | Yes | Yes |
| | CYP2C9 inhibitor | No | No | Yes | Yes | No | Yes | No |
| | CYP2D6 inhibitor | Yes | Yes | Yes | Yes | Yes | Yes | No |
| | CYP3A4 inhibitor | Yes | Yes | Yes | Yes | Yes | Yes | Yes |
| | log Kp (cm/s) | -5.31 | -6.06 | -5.03 | -4.94 | -5.06 | -5.9 | -5.91 |
| **Drug likeness** | Lipinski #violations | 0 | 0 | 0 | 0 | 0 | 0 | 0 |
| | Ghose #violations | 0 | 0 | 1 | 1 | 0 | 0 | 0 |
| | Veber #violations | 0 | 0 | 0 | 0 | 0 | 0 | 0 |
| | Egan #violations | 0 | 0 | 1 | 1 | 0 | 0 | 0 |
| | Muegge #violations | 0 | 0 | 1 | 1 | 0 | 0 | 0 |
| | Bioavailability Score | 0.55 | 0.55 | 0.55 | 0.55 | 0.55 | 0.55 | 0.55 |
| **Medicinal Chemistry** | PAINS #alerts | 0 | 0 | 0 | 0 | 0 | 0 | 0 |
| | Brenk #alerts | 0 | 0 | 0 | 0 | 0 | 0 | 0 |
| | Lead likeness #violations | 1 | 1 | 2 | 2 | 2 | 2 | 1 |
| | Synthetic Accessibility | 2.58 | 2.85 | 2.95 | 3.11 | 2.81 | 2.63 | 2.43 |
| **Toxicity** | Mutagenic | No | No | No | No | No | No | Yes |
| | Tumorigenic | No | No | No | No | No | No | Yes |
| | Reproductive Effective | No | No | No | No | No | No | Yes |
| | Irritant | No | No | No | No | No | No | Yes |

[1]Pyrimethamine, used as positive control. [2]Total polar surface area. [3]MS: Moderately soluble. [4]PS: Poorly soluble. [5]Gastrointestinal. [6]Blood-brain barrier. [7]P-glycoprotein substrate.

**Supplementary Material Table S3.** ADMET properties predictions for six marinoquinoline derivatives and pyrimethamine according to AdmetSAR.

| | MQ-1 | MQ-2 | MQ-3 | MQ-4 | MQ-5 | MQ-6 | PYR[1] |
|---|---|---|---|---|---|---|---|
| **Ames mutagenesis** | + | + | + | + | + | + | - |
| **Acute Oral Toxicity (c)** | III | III | III | III | III | III | II |
| **Androgen receptor binding** | + | + | + | + | + | + | + |
| **Aromatase binding** | + | + | + | + | + | + | + |
| **Blood Brain Barrier** | + | + | + | + | + | + | + |
| **BRCP inhibitor** | - | - | - | - | - | - | - |
| **BSEP inhibitor** | + | + | + | + | + | + | + |
| **Caco-2** | + | - | - | - | - | + | + |
| **Carcinogenicity (binary)** | - | - | - | - | - | - | - |
| **Carcinogenicity (trinary)** | Non-required | Non-required | Danger | Danger | Non-required | Non-required | Non-required |
| **CYP1A2 inhibition** | + | + | + | + | + | + | + |
| **CYP2C19 inhibition** | + | - | + | + | + | + | - |
| **CYP2C8 inhibition** | + | + | + | + | + | + | + |
| **CYP2C9 inhibition** | - | - | + | + | + | - | - |
| **CYP2C9 substrate** | - | - | - | - | - | - | - |
| **CYP2D6 inhibition** | + | - | - | - | - | + | + |
| **CYP2D6 substrate** | - | + | - | - | + | - | - |
| **CYP3A4 inhibition** | + | + | - | - | + | + | - |
| **CYP3A4 substrate** | + | + | + | + | + | + | - |
| **CYP inhibitory promiscuity** | + | + | + | + | + | + | + |
| **Eye corrosion** | - | - | - | - | - | - | - |
| **Eye irritation** | + | - | - | - | - | - | - |
| **Estrogen receptor binding** | + | + | + | + | + | + | + |
| **Glucocorticoid receptor binding** | + | + | + | + | + | + | + |
| **Hepatotoxicity** | + | + | + | + | + | + | + |
| **Human Ether-a-go-go-Related Gene inhibition** | + | + | + | + | + | - | - |

(cont.)

| | | | | | | | |
|---|---|---|---|---|---|---|---|
| **Human Intestinal Absorption** | + | + | + | + | + | + | + |
| **Human oral bioavailability** | + | + | + | + | + | + | + |
| **MATE1 inhibitor** | - | - | - | - | - | - | - |
| **Mitochondrial toxicity** | - | + | - | - | + | - | + |
| **Micronuclear** | + | + | + | + | + | + | + |
| **Nephrotoxicity** | - | - | + | + | - | - | + |
| **Acute Oral Toxicity** | 2.777612209 | 3.192592621 | 3.563766003 | 4.35090971 | 3.287763119 | 2.658835411 | 2.981128 |
| **OATP1B1 inhibitor** | + | + | + | + | + | + | + |
| **OATP1B3 inhibitor** | + | + | + | + | + | + | + |
| **OATP2B1 inhibitor** | - | - | - | - | - | - | - |
| **OCT1 inhibitor** | - | - | - | - | + | - | + |
| **OCT2 inhibitor** | - | + | - | - | - | - | + |
| **P-glycoprotein inhibitor** | - | + | - | + | + | - | - |
| **P-glycoprotein substrate** | - | + | - | - | + | - | - |
| **PPAR gamma** | + | + | + | + | + | + | + |
| **Plasma protein binding** | 1.003971338 | 0.955927551 | 1.032086134 | 0.9817757010000001 | 0.998464346 | 1.035541773 | 0.92094641 |
| **Reproductive toxicity** | + | + | + | + | + | + | + |
| **Respiratory toxicity** | - | + | - | + | + | + | + |
| **Skin corrosion** | - | - | - | - | - | - | - |
| **Skin irritation** | - | - | - | - | - | - | - |
| **skin sensitization** | - | - | - | - | - | - | - |
| **Subcellular localization** | Mitochon-dria | Mitochon-dria | Mitochon-dria | Mitochon-dria | Mitochon-dria | Mitochon-dria | Lysosomes |
| **Thyroid receptor binding** | + | + | + | + | + | + | + |
| **UGT catalyzed** | - | - | - | - | - | - | - |
| **Water solubility** | -3.237773778 | -2.510816114 | -5.165401196 | -5.326074509 | -3.462040937 | -3.853552013 | - |

[1]Pyrimethamine, used as positive control.
